# COVID-Scraper: An Open-Source Toolset for Automatically Scraping and Processing Global Multi-Scale Spatiotemporal COVID-19 Records

**DOI:** 10.1109/ACCESS.2021.3085682

**Published:** 2021-06-03

**Authors:** Hai Lan, Dexuan Sha, Anusha Srirenganathan Malarvizhi, Yi Liu, Yun Li, Nadine Meister, Qian Liu, Zifu Wang, Jingchao Yang, Chaowei Phil Yang

**Affiliations:** NSF Spatiotemporal Innovation CenterGeorge Mason University 3298 Fairfax VA 22030 USA; Department of Geography and Geoinformation ScienceGeorge Mason University 3298 Fairfax VA 22030 USA; Department of Aerospace and Mechanical EngineeringUniversity of Notre Dame 6111 Notre Dame IN 46556 USA; Department of PhysicsHarvard University 1812 Cambridge MA 2138 USA

**Keywords:** Web scraper, COVID-19, spatiotemporal data, multiple scale

## Abstract

In 2019, COVID-19 quickly spread across the world, infecting billions of people and disrupting the normal lives of citizens in every country. Governments, organizations, and research institutions all over the world are dedicating vast resources to research effective strategies to fight this rapidly propagating virus. With virus testing, most countries publish the number of confirmed cases, dead cases, recovered cases, and locations routinely through various channels and forms. This important data source has enabled researchers worldwide to perform different COVID-19 scientific studies, such as modeling this virus’s spreading patterns, developing prevention strategies, and studying the impact of COVID-19 on other aspects of society. However, one major challenge is that there is no standardized, updated, and high-quality data product that covers COVID-19 cases data internationally. This is because different countries may publish their data in unique channels, formats, and time intervals, which hinders researchers from fetching necessary COVID-19 datasets effectively, especially for fine-scale studies. Although existing solutions such as John’s Hopkins COVID-19 Dashboard and 1point3acres COVID-19 tracker are widely used, it is difficult for users to access their original dataset and customize those data to meet specific requirements in categories, data structure, and data source selection. To address this challenge, we developed a toolset using cloud-based web scraping to extract, refine, unify, and store COVID-19 cases data at multiple scales for all available countries around the world automatically. The toolset then publishes the data for public access in an effective manner, which could offer users a real time COVID-19 dynamic dataset with a global view. Two case studies are presented about how to utilize the datasets. This toolset can also be easily extended to fulfill other purposes with its open-source nature.

## I. Introduction

The worldwide COVID-19 pandemic has infected billions of people in the past year [1]. This global crisis triggered lockdowns in most countries around the world for months in hopes to slow the spread of this novel virus and save lives [2]. Inevitably, the normal lives of citizens have been heavily disturbed and impacted. Scientists all over the world are studying this pandemic to analyze the spreading dynamics, design effective control policies, predict the next possible outbreak centers, develop vaccines, and optimize vaccination strategies. COVID-19 virus samples, statistics of positive cases, existing policies, and environmental factors have become important data for COVID-19 related research [3]. Another example is spatiotemporal COVID-19 records, which most countries have gradually published through virus testing since early 2020. Collecting, organizing, and distributing spatiotemporal COVID-19 records provide avenues and data sources to support COVID-19 studies in different fields such as public health, economics, and environmental science. Governments and organizations of each country recognize the need for public records. For example, most of the COVID-19 cases data comes from international agencies (i.e. the World Health Organization (WHO) and the Global Health Council (GHC)), or individual national organizations (i.e. the Centers for Disease Control and Prevention (CDC) and the National Health Commission of the People’s Republic of China). These organizations have subcommittees that collect and produce datasets published to the public [4]. However, for researchers, one difficulty in obtaining these datasets is that information is published in various sources, formats, types, scales, channels, and time intervals by different countries. This makes it time-consuming to acquire the latest fused structured data for each country routinely, thus hindering the response progress to fight COVID-19. To address this problem, we developed the COVID-Scraper, a toolset for automatically aggregating the multiple sources of spatiotemporal COVID-19 dataset from different scales into one spatiotemporal framework with tailored data structures that benefit related studies.

For some actors, like large institutions, this task has been undertaken since the COVID-19 outbreak. John’s Hopkins is a prime example that provides a daily updated COVID-19 Dashboard by pulling data from eight different non-governmental sources, including the WHO, the CDC, the European Centre for Disease Prevention and Control (ECDC), and numerous countries’ data repositories and organizes the data into one dataset for public sharing [5]. However, the process of data collecting, organizing, and structuring for their “COVID-19 Dashboard by the Center for Systems Science and Engineering (CSSE) at Johns Hopkins University (JHU)” is not transparent, which leads to another challenge that some users cannot use it as a tool to acquire datasets from preferred data sources with customized data structures and setup user-defined acquisition frequency. Another widely known system is the 1Point3Acres COVID-19 dashboard, which has gained over 2.8 billion visits [6]. Similar to the COVID-19 Dashboard by JHU, users cannot customize the data sources for countries. Another issue is for a display dashboard, the raw data is difficult to access by the public (even it claims the data could be distributed with permission). Hence, it is impossible for users to define the granularity of data, filter the content of data and select the categories of data for customized scholar research. In other words, existing solutions are not flexible enough for users, especially those which have specific requirements to obtain targeted datasets.

We developed the COVID-Scraper as an open-sourced COVID-19 scraping toolset by adopting the technology of web crawlers to collect, filter, organize, pre-process, and store multi-scale spatiotemporal COVID-19 records for each nation over the world to generate a comprehensive data product in a single run. It is highly flexible and allows users to customize the data sources, data structures, filter criteria, database setup, and visualization formats with only minor adjustments. Once those parameters are set up, this toolset can be easily deployed on any cloud platform to fetch required COVID-19 spatiotemporal datasets automatically. In addition, the COVID-Scraper is easy to use and process data effectively. For example, it can finish acquiring the available COVID-19 datasets from all countries over the world within about six minutes. Furthermore, the COVID-Scraper works exceptionally well for countries that do not provide good, well-structured data from their official reports about their current situation of COVID-19 other than portable document format (PDF), or pictures in their reports. It can also be used as a powerful toolset for building historical spatiotemporal COVID-19 data records for some countries that only provide the latest COVID-19 data reports.

In this paper, the different types of spatiotemporal COVID-19 data sources from different countries consumed by the COVID-Scraper are discussed in section III. Then the components, mechanism, and implementation of this toolset are detailed in section IV and include: 1) a workflow of how the COVID-Scraper functions, 2) how it is designed to cater to different types of data sources, and 3) the processing of automation configurations. Section V details two case studies of how the scraper functioned and produced data for countries especially those that did not have well-documented information for easy access. Performance tests are conducted to demonstrate the overall performance of a single complete scraping process and processing time for different data types. We also introduce two cases that utilized the final data product generated by the COVID-Scraper to monitor the medical resource deficiency dynamics and the impact of social distancing measures on COVID-19 cases and mortality. The paper is concluded with discussions of the implications of the scraper and the future directions of the COVID-Scraper. The major contributions of this work are:

1) an open-sourced COVID-19 scraping toolset with web crawlers to collect, filter, organize, pre-process, and store multi-scale spatiotemporal COVID-19 records for each nation over the world.
2) a list of data scraping scripts to accommodate COVID-19 spatiotemporal data scraping tasks for various types of source data published by various countries.
3) a workflow that could automatically drive this scraping toolset and generate a comprehensive data product in a single run
4) an up-to-date multi-scale COVID-19 records data product is provided in GitHub repository and a cloud-based database for the public.
5) an operational dashboard is maintained to visualize the data product for quick query and access.

## II. Literature Review

Web scraping is a data mining technology that is commonly used for extracting unstructured data from different online sources and restructuring and converting acquired data into a structured form that can be further stored and analyzed in a database [7]. The benefit of a well-designed web scraper is that it automatically sifts through targeted data sources and form valuable information into a comprehensive dataset. There are different forms of web scraping including copy and pasting, text grabbing, HTML parsing, and others [7]. A benefit of web scraping is that it simulates human interaction with a web page and can obtain attribute data from the web page itself [8]–[10]. This is beneficial because it brings in pertinent information that is relevant to the topic assigned to look for and not scraping for erroneous information. For example, Weng and his colleagues applied web scraper techniques to collect large-scale datasets of horticultural products information to predict the trend of price fluctuation with Auto Regressive Integrated Moving Average (ARIMA) and integrated recurrent neural network (RNN) model [11]. Pawar and colleagues implemented a web scraper to search medicinal plants and relevant diseases in the India Ayurvedic system [12].

Web scraping is widely used by epidemiological research and public health studies. By scraping and analyzing text-based data from the Internet, researchers can successfully detect diseases and food hazards, as well as predict potential pandemics. For example, Pollett and colleagues used a web scraper as a tool to scrape unstructured Internet newswire data to timely detect outbreaks and epidemics from vector-borne diseases [13]. Walid and his team scraped worldwide Twitter data for 2 years [14]. By applying sentiment analysis and natural language processing on Walid’s data, they built a model to detect and predict cancer. In addition to diseases detection, web scraping has been adopted in food hazards detection and dissemination. By scraping the events related to food hazards from news and social media, Ihm and colleagues built a system to prevent and control food hazards in Korea [15]. In addition, Majumder *et al.* utilized web scraped data collected by HealthMap coupled with Google Trend time series data to calculate the R0 and predict the outbreak level of Zika virus in 2015 [16]. Beyond scraping text-based data from Internet resources, images have been scraped as a valuable dataset to support public health research. For example, Li *et al.* scraped illicit drug dealer-related photos and posts from Instagram. With 3 different deep learning models applied, they detected 1129 drug dealers successfully [17].

This same technique can be applied to COVID-19 related data collection. Chen *et al.* adopted a web crawler to collect emotion and experience data of online education platforms for users to assess the satisfaction and quality of online education under the pandemic [18]. La *et al.* scanned and collected official media news related to COVID-19 in Vietnam to evaluate the response from policymaking, social media, and science journalism regarding the outbreak [19]. Xu et al scraped Weibo posts from Wuhan, China at the early stage of the COVID-19 outbreak to analyze public reaction, knowledge, and attitude [20]. Their findings potentially support future policy making and possible future outbreak responses.

However, it is worthwhile to point out that an expressed concern in the field of web scraping due to the fact that scrapers can obtain personal information and publish it to an open database [21]–[23]. This becomes even more sensitive when medical records are retrieved by the scraper. In our study, the COVID-19 web scraper is aimed at collecting fine scale spatiotemporal COVID-19 records for countries that are releasing numerical data globally and aggregating them into a central database without directly working with the personal medical records.

## III. Data Types and Availability for the COVID-Scraper

**FIGURE 1.**
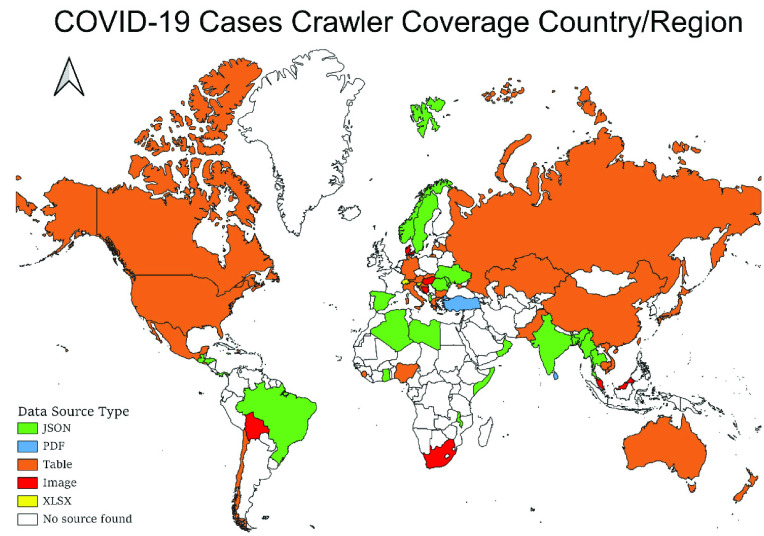
Global scale data availability and the COVID-Scraper coverage.

The COVID-Scraper was developed to automatically and routinely collect spatiotemporal COVID-19 records released by countries all over the world. However, there are varying degrees to which these records are available from different countries (Figure 1). Some countries such as the U.S. and China provide trustable, comprehensive, fully processed, ready to use datasets through official portals. These datasets are usually in Comma-Separated Values (CSV) tabular or JavaScript Object Notation (JSON) structured format that stored in a standalone file or cloud shared documents such as Google Spreadsheet [24]. Some other countries like Turkey and Chile also provide information on COVID-19, but it is not well organized. For example, the data may be published on a dynamic website inside a PDF file or embedded in an image-based file. In these contexts, the datasets cannot be read and parsed by text-based processing algorithms directly and automatically. Hence, advanced technologies should be developed and integrated to mine the expected dataset, extract required information from those unstructured data sources, and convert them into user-defined data structures for storage and sharing. Currently, the COVID-Scraper scans and scrapes all countries with available data sources daily (Figure 1). It will skip those countries without any available data source.

Countries listed in Table 1 are the major focus of the COVID-Scraper, which provides COVID-19 records in unstructured and not well-organized formats (Table 1). Our toolset checks the data sources to confirm availability before every run and reports exceptions if the data source is no longer valid or the data type/format has been changed.

**TABLE 1:** Major Countries and DATA Sources Scraped by the COVID-Scraper

Region	Data type	Data Source	Region	Data type	Data Source
Albania	JSON	National Agency for Information Society [25]	Algeria	JSON	Algerian Ministry of Health [26]
Australia	Table	Department of Health Australia [27]	Austria	Table	Federal Ministry Republic of Austria [28]
Bangladesh	JSON	Information and Communication Technology Division [29]	Bolivia	Image	Bolivia Segura COVID-19 [30]
Bosnia & Herzegovina	Image	Ministry of Civil Affairs of Bosnia and Herzegovina [31]	Brazil	JSON	Ministry of Health of Brazil [32]
Bulgaria	Table	Government of Bulgaria [33]	Cambodia	Table	Ministry of Health of Cambodia [34]
Canada	Table	Government of Canada [35]	Chile	Table	Ministry of Health of Chile [36]
Croatia	Table	Ministry of Health of Croatia [37]	Denmark	Image	Statens Serum Institute [38]
El Salvador	Table	Government of El Salvador [39]	Germany	Table	Robert Koch Institute [40]
Ghana	JSON	Ghana Health Service [41]	Greece	Table	Government of Greece [42]
Guatemala	JSON	Government of El Salvador [43]	Haiti	Table	Government of Haiti [44]
Honduras	JSON	Office of Communications and Presidential Strategy [45]	Hungary	Image	Government of Hungary [46]
India	JSON	Ministry of Health and Family Welfare of India [47]	Italy	Table	Civil Protection Department [48]
Jamaica	JSON	Ministry of Health and Wellness of Jamaica [49]	Japan	Table	Ministry of Health, Labour and Welfare of Japan [50]
Kazakhstan	Table	Ministry of Health of Kazakhstan [51]	Latvia	Table	Government of Latvia [52]
Lebanon	Table	Lebanese Ministry of Information [53]	Libya	JSON	National Center for Disease Control of Libya [54]
Mainland China	Table	National and provincial and municipal health committees [55]	Malawi	JSON	Ministry of Health of Malawi [56]
Malaysia	Image	Ministry of Health of Malaysia [57]	Mexico	Table	Government of Mexico [58]
Moldova	JSON	Ministry of Health, Labor and Social Protection of Moldova [59]	Myanmar	JSON	Ministry of Health and Sports of Myanmar [60]
New Zealand	Table	Ministry of Health of New Zealand [61]	Nigeria	Table	Nigeria Centre for Disease Control [62]
Norway	JSON	Norwegian Institute of Public Health [63]	Oman	JSON	Ministry of Health of Oman [64]
Pakistan	Table	Government of Pakistan [65]	Panama	JSON	Ministry of Health of Panama [66]
Romania	JSON	Government of Romania [67]	Russia	Table	The Russian Government [68]
Sierra Leone	Table	Government of Sierra Leone [69]	Slovakia	Table	Ministry of Investments, Regional Development, and Informatization of the Slovak Republic [70]
Slovenia	XLSX	Republic of Slovenia [71]	Somalia	JSON	Ministry of Health of Somalia [72]
South Africa	Image	Republic of South Africa [73]	Spain	JSON	Ministry of Health of Spain [74]
Sri Lanka	PDF	Ministry of Health, Sri Lanka [75]	Sweden	JSON	Public Health Agency of Sweden [76]
Switzerland	XLSX	Federal Office of Public Health of Switzerland [77]	Thailand	JSON	Department of Disease Control of Thailand [78]
Turkey	PDF	Ministry of Health, Turkey [79]	Ukraine	JSON	National Security and Defense Council of Ukraine [80]
United States	Table	USA Facts [81]	Vietnam	Table	Ministry of Health, Vietnam [82]

From a computing perspective, data types of COVID-19 records published by different countries are in structured or unstructured formats. CSV is one of the most commonly used formats for structured data. However, other formats are also adopted by official sources for releasing tabular cases data. For example, cases data from Brazil [32] are in the Microsoft Excel format (.xlsx), which will be required to be converted into CSV before further processing. JSON is another format for structured data, typically provided as standalone JSON files or via API by the data sources. In addition to structured data formats, unstructured data formats include original HTML, PDF, or images (jpg, png, bmp, etc.).

The COVID-Scraper is developed to accommodate various types of COVID-19 case datasets in structured or unstructured formats. In our study, open-sourced packages and browser rendering tools [83] have been applied to support scraping, parsing, and analyzing data in different formats. Once required spatiotemporal COVID-19 records are extracted from the data sources, the COVID-Scraper will filter, organize, and store the data into a single database under the same data framework. In section VI, the COVID-Scraper’s automation methodologies, structures, and detailed implementation will be discussed for each type of data from different countries.

## IV. Method

**FIGURE 2.**
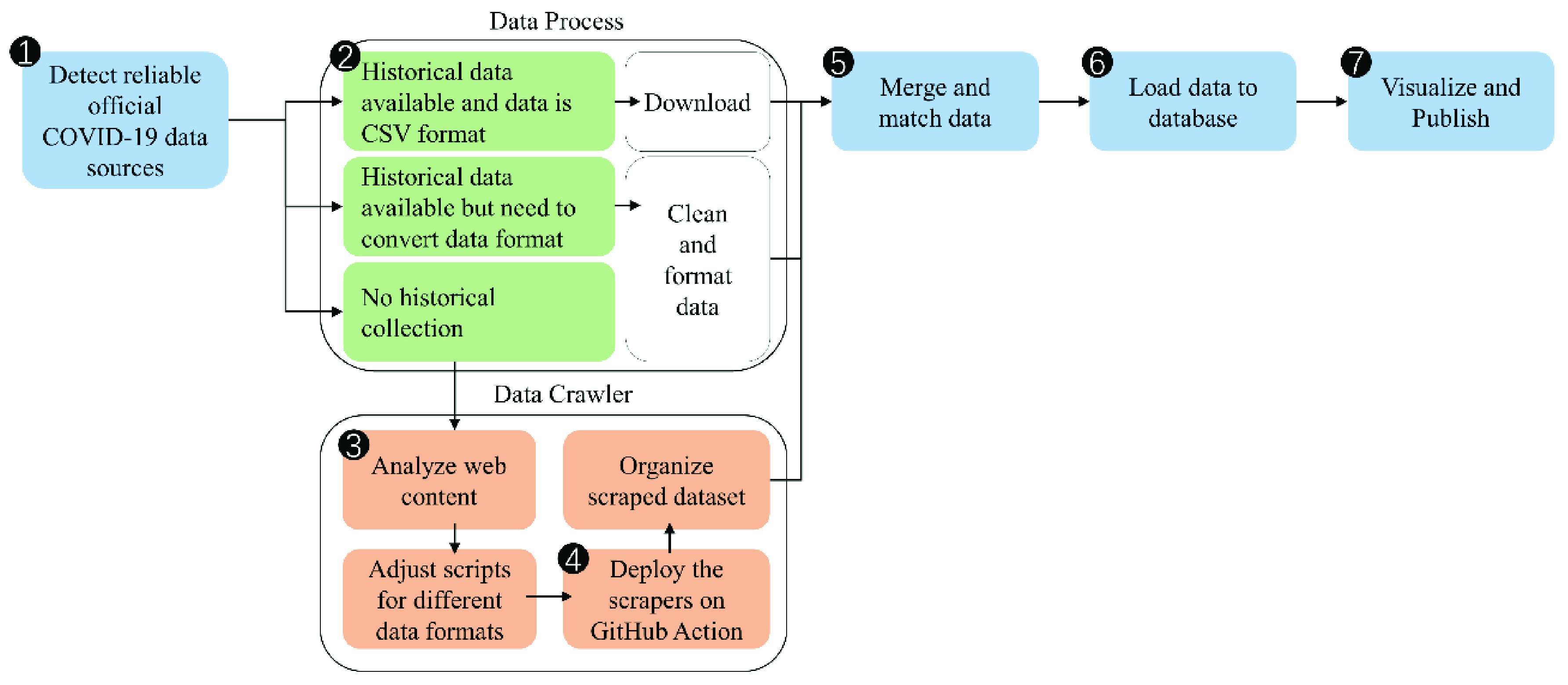
Overall workflow.

The overall workflow of the COVID-Scraper toolset contains seven steps (Figure 2):

1. Detecting the official, trust-worthy websites for COVID-19 spatiotemporal records data from each individual country. Choose a preferred data source for each target country.
2. Scanning all the targeted data sources and analyzing what type of data should be collected and extracted.
3. Adjusting template crawler unit to accommodate specific needs of each unique data source. Testing it and verifying that only the expected data are collected from the target data source.
4. Assembling all crawlers into a toolset and hosting it on a platform for automation. In our operational version, GitHub actions have been adopted for this purpose. By utilizing GitHub actions, a workflow was developed and configured, including managing scraping tasks, handling exceptions, and processing frequency to automatically run the COVID-Scraper on demand.
5. Fetching collected results from the configured temporary data store paths. Merging and matching those data based on unique geographical IDs. Unifying data structure based on user settings.
6. Verifying data quality and pushing them into a database as a data product.
7. Visualizing generated data product and publishing it as a web service for sharing, interactively viewing, and querying.

**FIGURE 3.**
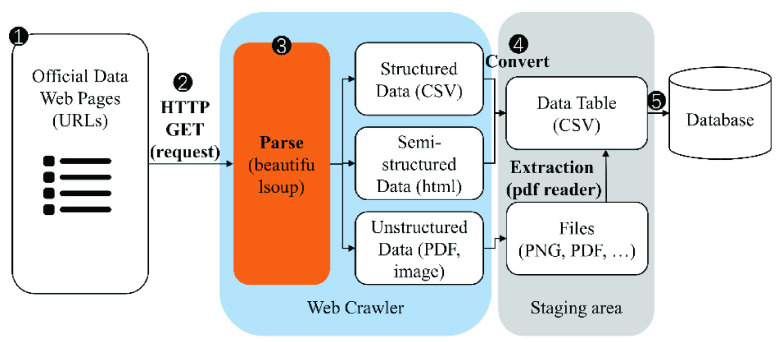
Methodology flow of COVID-Scraper.

From the perspective of algorithm implementation, HTTP requests will be sent at the initialization stage of the COVID-Scraper to all selected data sources (Figure 3). By parsing the acquired dataset in different formats via open-source packages, the spatiotemporal COVID-19 records from each country can be extracted. After all required datasets are collected, parsed, matched, and merged automatically, the whole dataset will be pushed into the database as a final data product.

To successfully accommodate various types of data sources, our toolset is designed to handle both structured datasets and unstructured datasets with only minor parameters adjustments.

### A. Structured Data Scraping

It is straightforward to handle most structured datasets because they are usually stored in wide or long formats. Long format tables contain columns corresponding to date, location, and numbers of confirmed/death/recovered cases. Since the long format is consistent with that of the daily report used in our database, the data are expeditiously processed by identifying the columns and matching the location names with ISO3, Hierarchical Administrative Subdivision Code (HASC), or local geographical IDs. Conversely, wide-format tables usually include multiple columns corresponding to different locations or dates, which must be converted to a long format before processing.

CSV, Microsoft Excel, and JSON are three major structured data types from COVID-19 cases data sources. If the records are provided in CSV format, they can be directly downloaded and sent for further processing. However, if these datasets are in Microsoft Excel or JSON, they have to be converted into CSV first before entering the next processing stage. Microsoft Excel format can be easily converted to CSV using the *pandas* package in Python. The JSON dataset, which is typically provided as standalone JSON files or via API by the sources, will require identifying the keys corresponding to date, locations, and case numbers from the JSON objects to convert them into tabular data format.

Occasionally, although the data is in a structured format, the link to the data file cannot be directly obtained. For example, one needs to click a button to download the data file from Brazil’s dashboard, where the link is not hardcoded in the source code but dynamically generated. For such cases, techniques to handle dynamic web pages will be adopted to obtain the download URL and acquire the expected dataset. The detailed implementation for handling dynamic web pages will be elaborated in the following section.

### B. Unstructured Data Scraping

Although structured data formats such as CSV and JSON are preferred, such data sources are not always available. Sometimes data must be scraped from web pages in addition to provided data links or APIs. Web pages can be developed in static and dynamic mode depends on the frameworks of websites, technology selection, and security concerns. In our toolset, both static and dynamic web pages can be scraped automatically.

#### 1) Static Web Pages Scraping

Static web pages are web pages with fixed content. When HTML data is loaded on the client’s web browser, it directly displays the same contents that are stored on the web server side. For static pages, an HTTP request is performed to retrieve HTML data from the web page. However, how to get required data out from web pages content effectively should be carefully considered. A challenge here is it will be very time consuming to design a parser and acquire valuable data when it encounters multiple layer nested web data structure in some web pages. Hence, it is recommended to apply an optimized approach to design parsers. Subsequently, various tools can be used to harvest the data from HTML content. For example, in our toolset, python packages “*requests”* and “*BeautifulSoup”* are used. The get() method in the *requests* package is used to send a GET request to the selected data source. After that, “*BeautifulSoup*” [84] is adopted to parse HTML, filter relevant HTML elements, and extract information from those elements. *BeautifulSoup* provides an object that represents web documents as a nested data structure. By searching and filtering required tags from this object, users can parse required information in straightforward ways, which saves significant amounts of time. Hence, the desired tag in the HTML page could be extracted using the *select* method in the *BeautifulSoup* package. Afterwards, the relevant information is stored as CSV files with proper settings.

#### 2) Dynamic Web Pages Scraping

Unlike static web pages with fixed data structure and web contents, some data on web pages are dynamically loaded with JavaScript and therefore they are not accessible in the requested HTML of the target web page. This results in a problem that by simply sending an HTTP request, web content cannot be fetched as expected. One way to scrape data from dynamic pages is to apply reverse engineering (i.e., identifying and manually analyzing JavaScript codes responsible for retrieving data). If relevant APIs can be identified, data could then be directly fetched through the APIs. For instance, ArcGIS is a commonly used technology to create many online COVID-19 dashboards. COVID-19 data published by those channels are normally hosted through ArcGIS’s feature server and can be queried through APIs. Those APIs share the same format, and once relevant information such as catalog instance ID and service name are pinpointed by inspecting the web page’s network activity, the corresponding ArcGIS query APIs can be obtained. In general, reverse engineering based on monitoring network activity can be used to find various other APIs.

However, this technique does not work smoothly sometimes especially when the relevant webpage code is minified and/or generated using a higher-level framework such as React.js, which makes the codes less readable. In those cases, HTML and Javascript codes need to be manually inspected to reverse engineer relevant information. To conquer this problem, headless browser rendering tools are adopted in our toolset to generate static HTML content for dynamic web pages. In the COVID-Scraper, *Selenium* web drivers are exploited to obtain rendered HTML content from dynamic pages. *Selenium* is a python package which is used to launch web driver from a remote machine. The *driver.get* method from *selenium* package is utilized to navigate to the selected data source. The drivers (such as *ChromeDriver*, *FirefoxDriver*) send direct commands to the corresponding web browser and retrieve the response. Occasionally, user input such as clicking on buttons and selecting relevant options from dropdown menus is necessary to obtain correct information, which is nicely supported by *Selenium*. To better integrate with the GitHub Actions workflows as mentioned before, remote web drivers are utilized by creating *Selenium* servers through Docker containers. Docker containers connecting to web services are natively supported by GitHub Actions, making the workflows much smoother. The generated HTML content can then be scraped as static web pages by using the methods described in section IV part B(1). The desired HTML tag in the page source is located by using the *find element by id* and *find element by css selector* methods in the *selenium* package.

#### 3) PDF Data Processing

In addition, it is common that some official COVID-19 daily reports are distributed as PDF documents by governments, which typically contains tables of case records. A challenge is to parse data directly from online PDF documents. After getting the required PDF documents back to a local server, extracting text-based information from the PDF file is also necessary.

In order to retrieve data from the PDF documents, two steps are applied in the COVID-Scraper.

1. The COVID-Scraper first gets links of the daily situation reports. Usually, there is an official web page containing links to all the reports. In such a case, the technique used for scraping from static web pages can be used to acquire the links. On occasion, documents of different dates share the same file name except for the date string. Thus, we can easily substitute the target date into the file name to obtain the link for the corresponding date.
2. After retrieving the links for PDF documents, several tools could be utilized to scrape data from the documents. Here we use *tabula-py*, a Python wrapper for *tabula-java*, which is a PDF table extraction engine. Normally, the relevant table contents are located at the same locations inside the PDF documents for different dates. Thus, coordinates of the areas containing those tables can be specified in *tabula-py* to obtain better results. The extracted data are then converted into CSV files for further processing. However, extra care needs to be taken to check the format and verify the data since sometimes the extraction output format may not be consistent.

#### 4) Image Data Processing

Another common format for distributing covid case records is as a picture, usually for easy understanding and easy share through social media. However, this will be a challenge for automatic web scrapers to get data directly. For this kind of data, we also use the python *BeautifulSoup* package to scrape those pictures with the specific ID or group name to fit users’ needs from static or dynamic websites. First, a *GET* request will be sent to the data source using *get*() method in the *requests* package. Then, the response of the request will be parsed by *BeautifulSoup*. Lastly, *select* method is applied to extract all image URLs from the data source to setup download tasks. After collecting pictures every day, our volunteers will manually record all the picture data to a CSV file.

Regardless of the format, typically data can be accessed via directly HTTP request or by reverse engineering. However, occasionally the data may be distributed in a platform that requires authenticated requests. For instance, the Philippines’ daily data are released on Google Drive. To access the data, client credentials need to be created for connecting to the Google Drive API before access to those specific resources.

In addition, source websites may have additional protection built to avoid DDOS attacks, which can also break the scrapers. For instance, the Croatia official COVID-19 website [37] utilizes the Cloudflare DDOS protection, and therefore requesting the source JSON file directly or via *Selenium* from a script will be denied. We use *FlareSolverr* to bypass the protection, which starts a proxy server and opens the requested URL via Chrome browser, and sends the requested file back after the Cloudflare challenge is solved.

### C. Data Collection Automation

**FIGURE 4.**
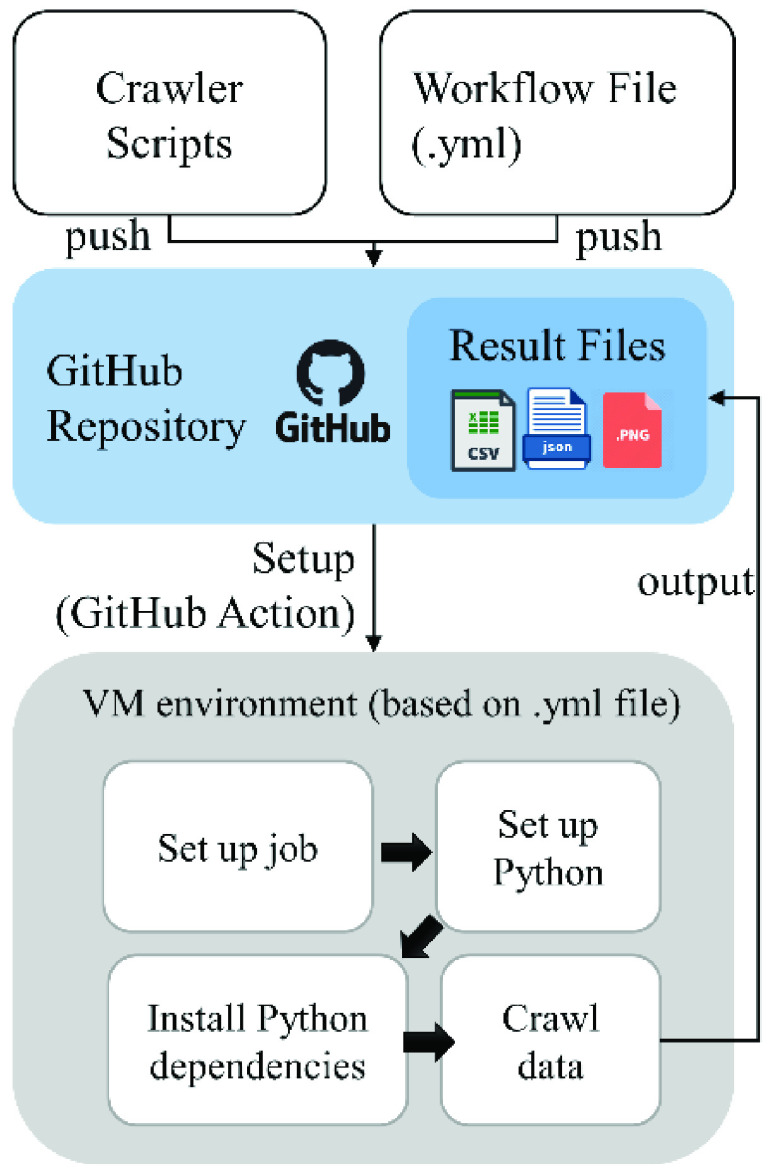
YML workflow to collect data automatically and routinely.

Once all crawler units are tuned properly, they can be assembled and processed automatically. Automation of the COVID-Scraper can be implemented in many different ways such as a simple script hosted on a server, automation toolkits, or workflows supported by cloud platforms. In our operational version, GitHub actions are applied to set up automated scraping processes in the COVID-Scraper. By hosting our toolset on GitHub actions and using the workflow files (.yml,.yaml) with a customized virtual environment, the COVID-Scraper can be built, deployed, and performed under manual control or operation by scheduled time and period (Figure 4).

The event-driven GitHub Action uses YAML file to define the parameters including 1) the event that triggers the workflow (parameter *on* is set to push event), 2) when to run the workflow (parameter *schedule,* which is set to run daily at 5:00 pm UTC in current operation), 3) the list of all the jobs that run in the workflow (parameter *job,* which is used to group together runs-on and steps parameters), 4) specify the configuration environment (parameters *runs-on* is set to Ubuntu Linux Runner in our case), 5) a group of all steps that needs to be run in the workflow (parameters *steps* is set to run the python environment in the runner and run the list of country-wise crawler scripts), and 6) the jobs to execute the command on the runner (parameter *run* is set to GitHub configuration settings to push the latest data). The *steps* parameter can be expanded to nest additional crawler scripts which in turn increases the total crawling time.

However, to ensure high quality dataset can be collected and saved locally before pushing them into a database, pre-configuration and post-processing will be performed to solve three possible issues:

• inconsistent location names in data sources
• inconsistent spatial scale
• temporal data gap

Those issues are nearly inevitable in practical operations. The mismatches of inconsistent names of administrative divisions, regions, or locations need to be fixed before collecting data. For instance, the Bogra District in Bangladesh officially changed its English spelling to Bogura District in 2018, but data scraped from Bangladesh’s COVID-19 dashboard [85] contains both spellings. Ignoring this issue may result in missing data or inaccurate cases count for some regions in those countries.

The inconsistent spatial scale and temporal data gap problem can be handled by post-processing. The truth is daily cases data for some countries may be reported for administrative divisions, health boards, or other statistical regions. In other words, after obtaining those datasets, the region names in those data are needed to match with HASC or local geographical IDs at consistent scales. For example, sometimes the data is reported at admin 2 (county/city) level while the required data scale is admin 1 (province/state) level [3], [86]. In such cases, a mapping table will be created to convert the admin 2 level dataset to admin 1 level. In the meantime, the cases records based on the admin 2 level will be aggregated and matched based on admin 1 regions. In addition, data may be missing for certain dates in some cases. For example, Denmark does not report daily cases on weekends. To make sure that the output reports are in consistent format, missing data are filled using data of the closest previous date when data are available. After the global dataset has been cleaned and formatted following each scraping, the cases dataset is exported as a CSV file. For each region, the corresponding record includes region name, country name, ISO3, HASC or local ID, and numbers of confirmed cases, deaths cases, and recovered cases when available. However, a data quality verification and validation will be done before pushing them into the database for effective inquires.

### D. Data Quality Control

Because the various data formats from the datasets collected globally, dealing with the instability of raw data quality is a challenge for automatically processed crawlers. For example, the structure of content from many sources is updated frequently which usually results in unexpected scraping errors. Therefore, detecting errors and anomalies is essential for this toolset. In addition, to quickly respond to errors during the toolset running, it is important to validate the collected data after the scraping process to make sure all datasets are correct and accurate. Three dimensions of data quality were evaluated in the automatic detection script, including data integrity; consistency; and validity. The completion check of continuous time-series data availability is required by data integrity. For consistency, the scraped data should be consistent with the sources. And several numeric rules were made for data validity evaluation. For example, 1) the accumulated viral case value should be unabated by time change; 2) the summarized cases value of a certain region should change among continuous time step; 3) a surge increase of new cases will be identified as abnormal growth; and 4) the accumulated case value of confirmed cases should be much larger than cases of death/recovered. To implement data quality evaluation in this toolset, validation scripts were developed as a component of the COVID-Scraper to compare each record from scraped data sources with corresponding data in validation data sources automatically. This process will be started after the crawling process. A data quality report will be produced to help verify if there is any inconstancies or mistakes in the collected dataset. For instance, the data of Nigeria is scraped from a public dataset [62] that provides admin1 level records. In the meantime, another dataset provided by Nigeria Centre for Disease Control is applied as a validation data source to ensure the accuracy of scraped dataset. By daily comparison of each pair of records in both datasets with the COVID-Scraper validation process, all mismatching and data gaps can be found before data finalization. The crawler for this specific country may need to be adjusted or the scraped data source may be replaced if any problems were detected during validation.

The current validation approach can accurately support data that has been formatted as CSV tabular format. However, for datasets extracted from PDF types, even if text recognition tool is applied, the recognition accuracy cannot be fully guaranteed. In those cases, a group of volunteers is helping manually check all the image type data daily, to make sure the data that has been published is in a high-quality standard.

### E. Final Data Product Generated by Data Scraper Toolset

**FIGURE 5.**
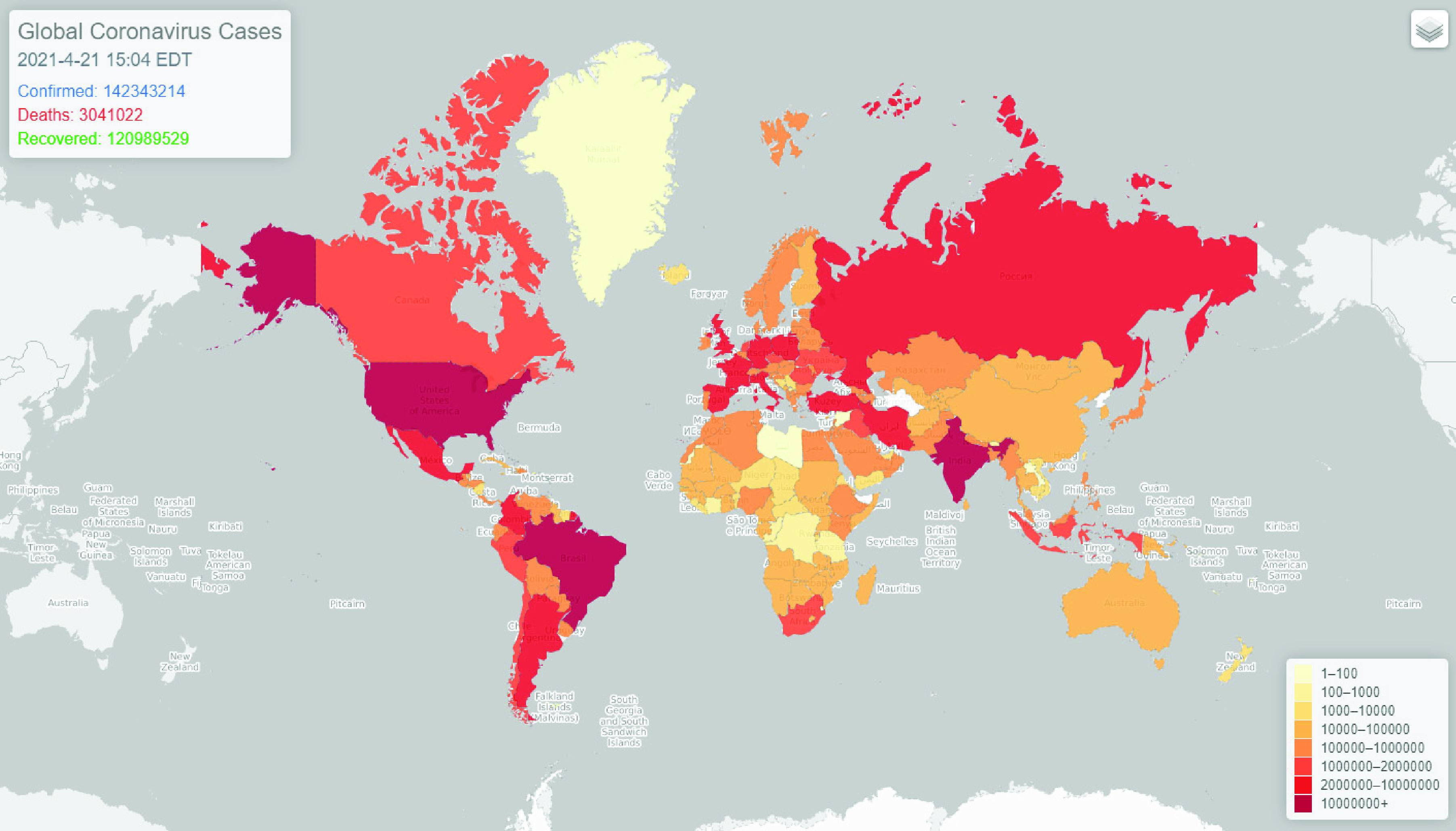
An operational dashboard of global COVID-19 records.

Once all scraped datasets pass the daily data quality check process, they will be converted into a standard table format joint with a basemap which serves as the spatial supplement attribute. The datasets are organized by region areas scaling from country level globally to admin 1 level of each country. Underneath each region area, daily reports, and time-series summary tables of confirmed, death, and recovered cases are produced and presented. After that, the COVID-19 data collection is pushed and shared via the GitHub repository [87] as the final data product with daily updates. In addition, the obtained data is also being loaded into a pre-designed relational database for backup and public representation purposes. An operational dashboard [88] has been developed and published online to represent and share the real-time global scale COVID-19 records in a visual manner with five minutes updating intervals by using the dataset from the database (Figure 5).

## V. Experiments and Discussion

To verify if the COVID-Scraper can work as designed to scrape COVID-19 dataset from different countries with various data formats, two study cases are selected in this section to represent the capability of our toolset to collect both structured data and unstructured dataset from static and dynamic web-based sources. Furthermore, performance is tested to check if the COVID-Scraper can be applied to scrape global datasets in a reasonable time thus support near real-time updating of the data product. After that, two study cases using the data product are introduced.

### A. Collecting From Chile Offcial COVID-19 Website

**FIGURE 6.**
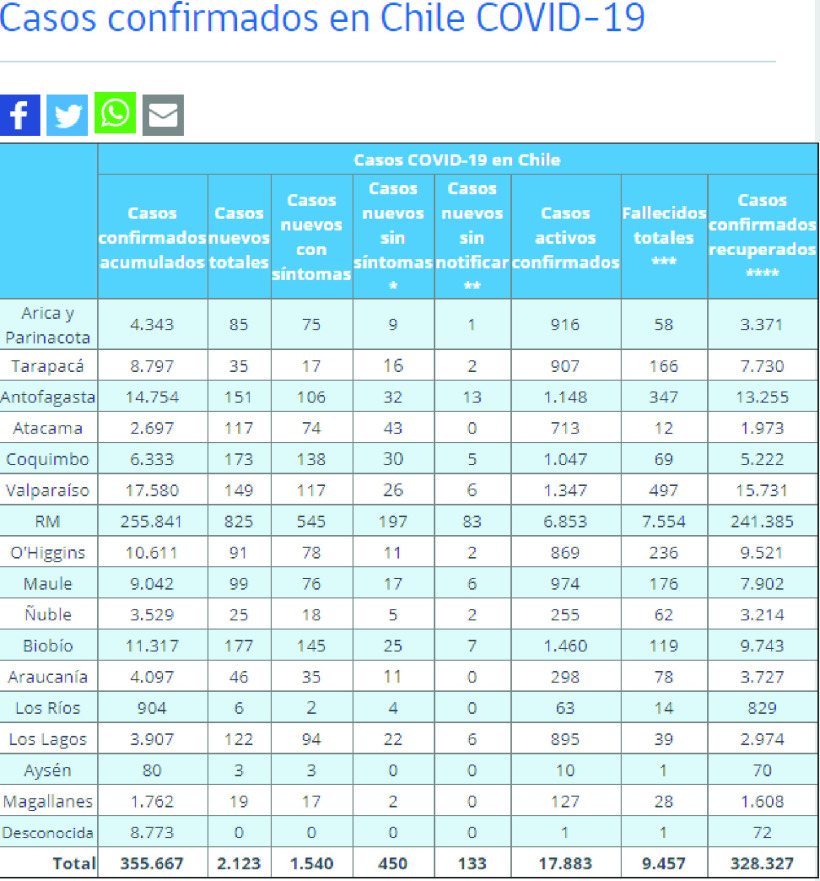
An operational dashboard of global COVID-19 records.

The COVID-19 dashboard of Chile [36] is an example of a static website (Figure 6). This website updates daily with the newest information about COVID-19 in Chile, all of which is shown as a table on the webpage.

To accommodate static websites, the key task is to parse the web elements and get required data from nested web structures. Three steps were applied:

1. Utilize the *BeautifulSoup* package in python to find the required data which are in the table or in <tr> or <td> html elements.
2. Apply *pandas* package to extract the required information from each parsed web element.
3. Concatenate all the data to a single CSV file as a result.

Once this file is created, it will be saved as a temporary result file and passed to a folder which is named by the time of the crawling process started. This experiment demonstrates successful functionality of COVID-Scraper, namely locating and scraping the datasets published by static websites. Scraped data has been stored in both database and the GitHub repository after the scraping process finished.

### B. Collecting From Pakistan COVID-19 Dashboard

**FIGURE 7.**
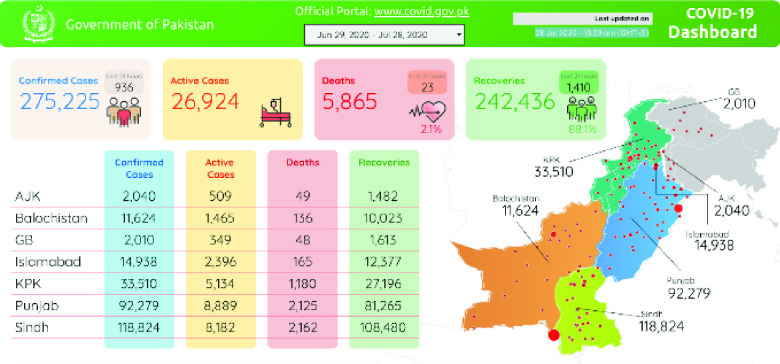
Pakistan COVID-19 Dashboard.

Pakistan’s COVID-19 dashboard [65] is an example of dynamic web page (Figure 7). In this website, daily cases data from seven top-level regions in Pakistan are displayed in a table located at bottom left of this dashboard page. However, the table is generated dynamically using Google Data Studio, hence the data cannot be scraped directly from the page’s HTML source code.

To solve this problem, four steps are needed before scraping data.

1. Analyze the network activity. The direct link of the dashboard in Google Data Studio (https://datastudio.google.com/embed/reporting/1PLVi5amcc_R5Gh928gTE8-8r8-fLXJQF/page/R24IB) should be detected by using browser tools such as Google Chrome’s developer tools.
2. Render the dashboard using *Selenium* web driver, which connects to and retrieves data from a web browser as discussed in section IV part B.
3. Start a standalone Selenium service on port 4444 to listen to incoming requests by adopting Github Actions’ service container capability.
4. Connect to the web driver at http://localhost:4444/wd/hub once the service in step 3 is established.

Until now, the web page is rendered and returned from the Selenium web driver. HTML elements in the rendered HTML document can be located using various methods provided by the web driver. By using those methods, such as identifying elements by CSS selectors, cell elements in the table that contain region names and cases data can then be identified. Each day, daily cases data is scraped and saved in a new file in CSV format. Data update time can also be extracted from rendered HTML as highlighted in Figure 7, as the temporal information. This experiment shows that COVID-Scraper can successfully scrape data from dynamic website. Different from static websites, web drive technologies have been adopted here to make sure targeted data can be recognized, accessed, and scraped.

### C. Performance Test

**FIGURE 8.**
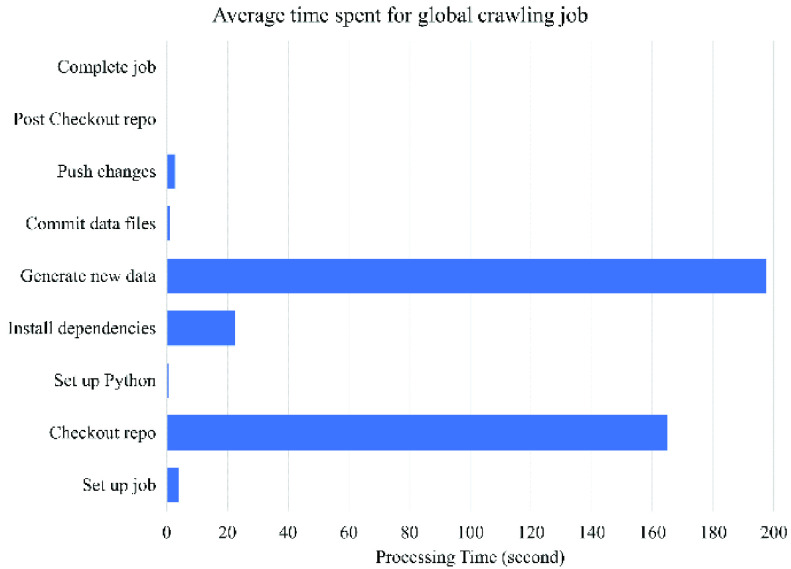
Overall performance of the COVID-Scraper.

To test if the COVID-Scraper can process the scraping tasks in a reasonable time for supporting COVID-19 related research, comprehensive performance tests are conducted. For the overall performance of the automatic scraping all available countries over the world, the average time spent for the whole GitHub action job is around six minutes fifty-five seconds by averaging 15 times tests (Figure 8). For each test, the processing time varies, mainly because the internet speed is unstable. When COVID-Scraper is starting, the process of setup job, setup Python, commit, and push result takes around 3 to 10 seconds to finish, which is quick. The major time-consuming steps are processing checkout repositories, installing Python dependencies, and generating new data, which are heavily impacted by the Internet speed during the processes. In addition, we noticed that after the source websites add more content or change the layout of their websites, the time spent crawling this website takes longer, or in the worst case, stops working. Once the scraper detects those abnormal statuses, a notification will be alarmed automatically to operators that support them to take action in real time. We continue to maintain and support this project in the long run to make sure it is working normally and effectively.

**FIGURE 9.**
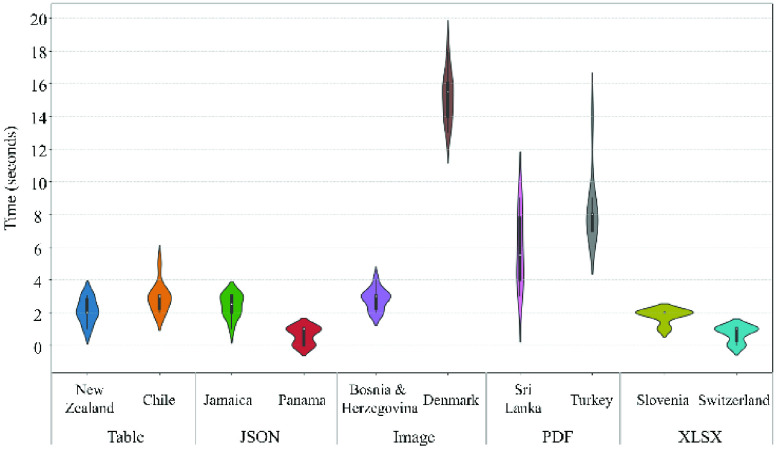
Performance tests on single countries with different data types.

To understand the detailed performance of the COVID-Scraper to get data from different countries, 10 countries have been selected including Austria, Chile, Jamaica, Panama, Bosnia, Hungary, Sri Lanka, Turkey, Slovenia, and Switzerland. Every two of those countries share the same data type, hence the five types of data scraping performance could be tested. Every country was run 15 times and the average time is calculated to reduce randomness. Austria and Chile publish data in table format. The average processing time is 2.0 and 2.9 seconds, respectively. Though they are in the same format, the reason for the difference in processing time is primarily due to the difference in size of crawling data. For Austria, the data size is 1. 1 KB whereas for Chile the data size is 1.46 KB. This may be the reason it takes more time to process Chile’s data in comparison to Austria’s data. In addition, the downloading speed during processing time also contributes to the difference. The Jamaica’s and Panama’s data are in JSON and show an average time of 2.4 and 0.6 seconds, respectively. Similar to Austria and Chile, the JSON file size of those two countries is the major reason for the time difference. The file size of Jamaica and Panama are 584 KB and 245 KB, respectively. Bosnia and Hungary publish data in image format and take an average time of 2.8 and 4 seconds. The file size of Hungary is greater than Bosnia which contributes to more processing time for Hungary. The data source of Sri Lanka and Turkey are in PDF format. The difference in processing time between those two countries is primarily due to two reasons. First, for Sri Lanka, the crawling script directly scrapes the data from the current data PDF file. But for Turkey, the script first crawls the HTML page to retrieve the latest PDF file link which then scraps the desired data from the PDF, which takes more time to process. Second, the required data of Sri Lanka is on the first page of the published PDF file whereas for Turkey, the desired data is on the third page during our performance testing which results in need of crawling more pages than Sri Lanka. The Slovenia and Switzerland data source are in XLSX format with a file size of 47.7 and 35 KB, respectively. The processing time for Slovenia is more than Switzerland because the file size is larger. Hence, the downloading time increases, causing an increase of processing time. To sum up, the processing time for countries mainly depends on the complexity of published website or data files, size of the data sources and, Internet speed.

### D. Use Cases With Scraped Data Product

The data generated by the COVID-Scraper has been used to support much scientific research within the academic community. Two studies are introduced here by applying the data generated by COVID-Scraper as one of the major data sources.

#### 1) Medical Resource Deficiency Dynamics

Since late March 2021, over 61 million of the U.S. population has been tested for a positive result for COVID-19. Whether medical resources were enough to handle the worst scenario amid the crisis is discussed and evaluated for public good. Three elements including ventilators, ICU beds, and critical medical care staff were reported as the fundamental medical resources to support critically ill patients. In this study, authors have created a medical resource deficiency index (MRDI) by using the COVID-Scraper data product and related COVID-19 medical data to measure the reality of the medical burden by using the crawled confirmed, death, recovered, and hospitalized viral cases at the county level in U.S [89].

MRDI is defined as the division of daily active cases and medical resources at the county scale, while the daily active cases refer to the difference of accumulated number of confirmed (positive tested) patients with accumulated number of deaths. And the medical resources were calculated by the number of licensed beds multiplied by the total number of critical care staff, specifically for COVID-19 response. The higher the value of MRDI, the medical source for a certain area is pressed harder. The accumulated viral case numbers of positive confirmed and deaths were extracted from USA Facts and cross-validated with sources from John Hopkins University. Hospital licensed bed number and critical medical care staff with comprehensive specialty were accessed from Definitive Healthcare consulting services and National Provider Identifier Registry (NPI) database respectively. All data collected in this study was converted into county scale with a unique identifier of county code by Census standard.

**FIGURE 10.**
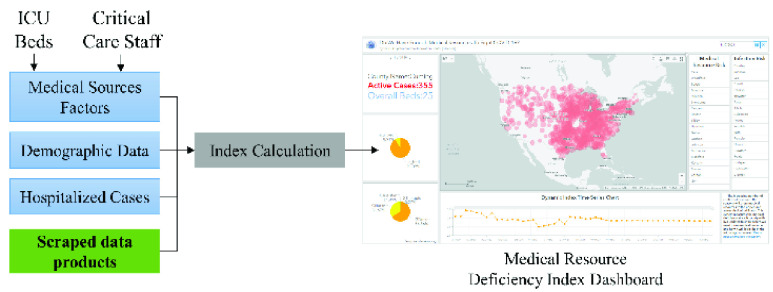
Use scraped data product to monitor medical resource deficiency dynamics of COVID-19.

To monitor and share the dynamic heterogeneity information of medical resource distribution, a medical resource deficiency dashboard is created based on the ArcGIS dashboard for analyzing and visualizing the generated results (Figure 10). A bubble map in the center of the dashboard represents the spatial distribution MDRI, where the area of circle refers to the index value. Two lists of counties are displayed on the right to show the statistics rank of MRDI and Infection Risk/Rate, which is interactively generated based on the selected extend of the map. An indicator and two pie charts (fraction of hospital bed types and medical care staff) are applied to display for each county on the left of the dashboard. To track the temporal pattern of the index, a line chart is built in the bottom to demonstrate the time-series analysis result for the selected area(s).

#### 2) The Impact of Social Distancing Measures on COVID-19 Cases and Mortality

**FIGURE 11.**
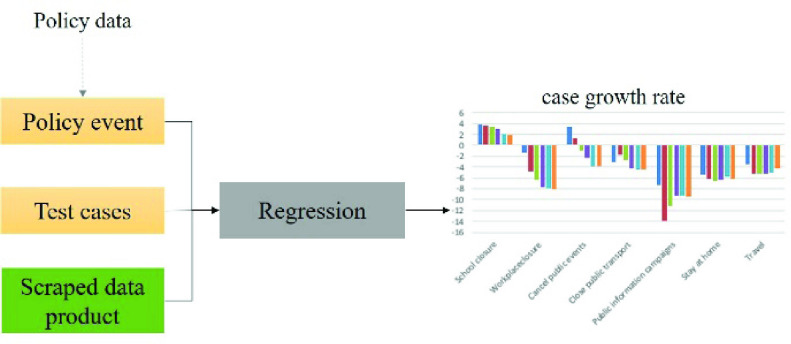
Use scraped data product to support COVID-19 policy analysis.

Another study was on the impact of control policies by using the COVID-Scraper data and corresponding policies dataset. In this study, authors analyzed a series of social distancing policies including school closure, workplace closure, cancellation of public events, public information campaigns, cancel public transport, internal movement restriction, and travel control that have been implemented to combat the worldwide pandemic. Previous studies have found social distancing policies are effective in mitigating COVID-19 [90], [91], however, these policies have negative impacts on economic development and normal life [92]. Limited understanding of the effectiveness of each individual policy has posed grand challenges on the reopening process in which the stringency of social distancing is reduced to balance health and development. A study investigating the effectiveness of seven major social distancing policies in the US on COVID-19 case and mortality growth rate [93] was conducted using the case data collected and policy data shared by the oxford policy tracker project [94]. To estimate the temporal dynamic impact of policies on the COVID-19 cases, policy data was transformed to 0-1 variables, which represent the policy’s implementation periods including one week, two weeks, three weeks, one month, two months, and more than two months. The scraped daily cumulative case data were converted to daily case growth rate, which is the difference between the logarithms of cumulative case numbers in two successive days. These six implementation indicators were regressed to case growth rate using panel regression analysis. Panel regression is widely used to analyze two-dimensional panel data which typically cross sectional (e.g., states, countries) and longitudinal (e.g., year, month) dimensions [95]. Specifically, a fixed effects panel regression model was adopted in our study, it could model unobserved heterogeneity through state-specific fixed effects [96]. In addition, the growth rate was multiplied by 100 in the regression, thus the regression coefficient of policy could be interpreted as percentage point changes of growth rate (Figure 11).

The study demonstrated that the stay-at-home orders, workplace closures, and public information campaigns can drastically decrease the confirmed case growth rate. Stay-at-home orders and workplace closure decrease case growth rate through changes in mobility while public information campaign impact confirmed case growth rate through channels other than mobility. In addition, regarding death case growth rate, stay-at-home orders and international travel controls had limited mitigation effect. The relation between policies and case growth rates learned by the study could provide policymakers a better understanding of the effectiveness of each policy to support decision-making.

## VI. Conclusion

The COVID-19 outbreak has impacted billions of people over the world. Governments, organizations, and research institutions are conducting rapid research on COVID-19 related problems that aim to bring people of every country back to normalcy. Detailed spatiotemporal COVID-19 records data is proved to be important evidence to support COVID-19 related research. However, how to collect, aggregate, store and share the data published by each country in the world to the community effectively is a challenge. To solve this problem, the COVID-Scraper was developed as an open-sourced toolset that can automatically scan, extract, collect, filter, refine, unify and store the public spatiotemporal COVID-19 records of fifty-eight countries around the world, which provide available COVID-19 data sources [97]. With minor code adjustments, this toolset can accommodate various types of data published by each country in various data formats, scales, channels, and publish frequencies. More importantly, for the countries that do not provide access to historical COVID-19 data, it can automatically build historical data collections to support research repeatedly on a certain frequency. The COVID-Scraper processes in a high effective manner by collecting data from countries over the world within a single run in about six minutes. After post-processing and data cleaning, the fetched data is unified and saved into a database for sharing. With daily data quality checking and data product production, a global COVID-19 data Github repository has been maintained since March 2020. In addition, a visualization component is developed in the COVID-Scraper to publish the data product as a web service for public view and access.

The COVID-Scraper utilized the web scraping technologies that are used in data science and GIS-related fields. By integrating open-source packages and tools for data extracting, network simulation, file, image parsing, and workflow automation, the COVID-Scraper is a highly flexible and automatic toolset that can process tasks unsupervised under users’ settings. With the nature of open source, users can easily customize the data sources, the data structure of the output data product, execution logic, processing frequency, and exception handling. In addition, users can modify the source code to extend it for collecting datasets for other purposes to support wider studies and tasks such as emergency response and natural disaster detection for saving lives.

Currently, a limitation is that the data quality control and validation cannot be fully automated because the accuracy of parsing and text extracting cannot be always guaranteed by using current packages. Hence, users need to intervene in the data quality control process for PDF and image type data to make sure the data product is of high quality. With the rapid development of text parsing from images, we will keep updating this component to minimize the human intervention in the automation process.
